# From Theory to Practice: Viewpoint on Economic Indicators for Trust in Digital Health

**DOI:** 10.2196/59111

**Published:** 2025-01-15

**Authors:** Felix Gille, Laura Maaß, Benjamin Ho, Divya Srivastava

**Affiliations:** 1 University of Zurich Digital Society Initiative Zurich Switzerland; 2 University of Zurich Institute for Implementation Science in Health Care Zurich Switzerland; 3 SOCIUM Research Center on Inequality and Social Policy University of Bremen Bremen Germany; 4 Leibniz ScienceCampus Digital Public Health Bremen Germany; 5 Department of Economics Vassar College Poughkeepsie, NY United States; 6 Department of Health Services Research Management, AI and Digital Health Lab (Centre for Healthcare Innovation Research) City St George’s University London United Kingdom; 7 Department of Health Policy London School of Economics and Political Science London United Kingdom

**Keywords:** trust, economics, digital health, digital health innovation, artificial intelligence, AI, economic evaluation, public trust, health data, medical apps

## Abstract

User trust is pivotal for the adoption of digital health systems interventions (DHI). In response, numerous trust-building guidelines have recently emerged targeting DHIs such as artificial intelligence. The common aim of these guidelines aimed at private sector actors and government policy makers is to build trustworthy DHI. While these guidelines provide some indication of what trustworthiness is, the guidelines typically only define trust and trustworthiness in broad terms, they rarely offer guidance about economic considerations that would allow implementers to measure and balance trade-offs between costs and benefits. These considerations are important when deciding how best to allocate scarce resources (eg, financial capital, workforce, or time). The missing focus on economics undermines the potential usefulness of such guidelines. We propose the development of actionable trust-performance-indicators (including but not limited to surveys) to gather evidence on the cost-effectiveness of trust-building principles as a crucial step for successful implementation. Furthermore, we offer guidance on navigating the conceptual complexity surrounding trust and on how to sharpen the trust discourse. Successful implementation of economic considerations is critical to successfully build user trust in DHI.

## Introduction

Public trust is a cornerstone of successful health systems [1,2]. Public trust is a key enabler for the introduction of trustworthy health data spaces for sharing and using health data in research, national electronic health record systems and the introduction of artificial intelligence (AI) systems in medicine. In 2023, the World Health Organization (WHO) in the European Region stated that 77% of its Member States believe the lack of trustworthy sources to access effective medical apps is a substantial implementation barrier to digital health transformation: “Trust is paramount – trust in technology, trust in data protection and, most critically, trust in the respect and safeguarding of privacy rights pertaining to personal health data” [3]. Trust in public health systems legitimizes these activities. Public trust is predicated on the belief that those systems provide a net benefit, where benefits include individual benefit, societal benefit, and health system benefit [4]. The increasing discourse on trust and trustworthiness of digital health systems interventions (DHI) has been met with an increase in nonbinding professional guidelines and codes of conduct. These guidelines are designed to guide users toward the ethical and trustworthy use of DHIs. Adhering to such guidelines can help foster public trust which, in turn, can foster their practical adoption. Some purposefully sampled guidelines in the area of AI as a prominent example of DHIs are highlighted in Table 1. The guidelines were selected to make sure national and international as well as government and nongovernment organizations were represented but we fully acknowledge that these reflect only a subset of the guidelines that exist.

**Table 1:** Overview of example guidelines about the trustworthy use of health data and artificial intelligence in relation to digital health system intervention.

Guideline	Providing organization	Context	Relevance for trust in digital health
Ethics guidelines for trustworthy Artificial intelligence [5]	European Commission	Strengthening national systems (including the health system) through artificial intelligence	“Trustworthy AI technologies can be used – and are already being used – to render treatment smarter and more targeted, and to help preventing life-threatening diseases. Doctors and medical professionals can potentially perform a more accurate and detailed analysis of a patient’s complex health data, even before people get sick, and provide tailored preventive treatment. […]”
Digital Trust Framework [6]	World Economic Forum	Companies willing to commit to earning digital trust of citizens and customers	“A technology provider’s reliability and security can have a significant impact on individuals’ safety, including on their physical or mental health. […]”
Global strategy on digital health 2020-2025 [7]	World Health Organization	Strengthening national health systems through digital health technologies	“The appropriate use of digital health takes the following dimensions into consideration: health promotion and disease prevention, patient safety, ethics, interoperability, intellectual property, data security (confidentiality, integrity, and availability), privacy, cost-effectiveness, patient engagement, and affordability. It should be people-centred, trust-based, evidence-based, effective, efficient, sustainable, inclusive, equitable and contextualized. […]”
Guidance on the rights-based and ethical use of digital technologies in HIV and health programs [8]	United Nations	Human rights, technical, and ethical considerations for countries adopting digital technologies for health and HIV	“A core component of successful HIV responses, trust is also a key element in the adoption and success of digital technologies for health. Without trust, the implementation and uptake of digital health will be weak, even if all other aspects of infrastructure, as well as legal and regulatory frameworks, are effective. Trust must be built for all types of relationships related to digital health – between patients and health-care providers; within the health sector institutions; between the State and its residents; and between a State, its residents and the private sector. […] Data breaches violate an individual’s right to privacy and erode trust in the health-care system. As technology evolves and health systems become more complex, the likelihood of data breach occurrences increase. Health systems should invest in information security and keep up to date on the latest in data protection to prevent breaches.”
A guide to good practice for digital and data-driven health technologies [9]	United Kingdom Department of Health and Social Care	Guiding document on “good practice” for digital health technology innovators in the United Kingdom	“When building an algorithm, be it a stand-alone product or integrated within a system, show it clearly and be transparent of the learning methodology (if any) that the algorithm is using. Undertake ethical examination of data use specific to this use-case. Achieving transparency of algorithms that have a higher potential for harm or unintended decision-making, can ensure the rights of the data subject as set out in the Data Protection Act 2018 are met, to build trust in users and enable better adoption and uptake.”
Code of Ethics for Data-Based Value Creation [10]	Swiss Alliance for Data-Intensive Services	Companies and organizations that offer products or services based on data	“Data management is networked. The quality of companies‘ data-based products and services depends in many ways on other organisations. Accordingly, it is important that in this network of interdependence that companies can trust each other. […] Transparency requires ensuring that customers are informed about all aspects of data handling – storage, management, and protection – thus creating (well-placed) trust.”

Such guidelines cover a range of trust-building principles, for example, the need to implement accountability mechanisms, security, or privacy measures, the importance of public and user-centered communication strategies, and the appropriate implementation of the regulation. One example is the Digital Trust Framework published by the World Economic Forum [6] which identifies principles along 8 dimensions: cybersecurity, safety, transparency, interoperability, auditability, redressability, fairness, and privacy. These principles aim to foster security and reliability, accountability, and oversight, as well as inclusive, ethical, and responsible data use for DHIs. The guiding principles are usually at an abstraction level that allows transferability across different contexts, necessitating careful tailoring during the implementation process. In practice, however, resources are limited. How do we determine which trust-building principles to prioritize, and which digital health policies should take precedence, especially when guidelines conflict? These guidelines are typically silent on the economic implications of trust-building, which could provide guidance on the costs and benefits of implementation. A clear need for economic guidance persists. This observation is underscored by anecdotal observations made by FG in Switzerland in the past few years during workshops and meetings discussing trust in DHIs.

After attending an expert workshop aiming at identifying a list of trust-building principles comprising a trustworthy data space, a fellow participant asked: “This list looks convincing, but I am the only IT (Information Technology) security employee in a small firm, we have limited resources, so which of the principles are cost-effective?” (Workshop on trustworthy behavior, Bern, Switzerland)

While exploring a research collaboration with an industry partner where we aimed to test the performance of a nonbinding trustworthiness codex, the industry counterpart asked: “If our company adheres to a non-binding trustworthiness codex, isn’t there a risk that we face a competitive disadvantage compared to our competitors that do not commit to these measures but still adhere to the present legislation?” (Business meeting on the performance of a nonbinding trustworthiness codex, online, Switzerland).

Discussing trustworthiness principles to conceptualize Digital Trust, workshop members from the industry suggested that what we have today is robust conceptual evidence, but we clearly lack implementation experience and evidence showing what trust-building measures are costeffective. (Workshop exploring digital trust, Zurich, Switzerland).

When it comes to the implementation of trust-building measures and the economic evaluation of their effectiveness, we stumble upon a yawning knowledge gap. Three important unanswered questions require our attention: What are the costs of implementing trustworthiness guidelines? With limited resources at hand, which measures are cost effective? What instruments exist to gauge success?

We argue that the failure to address these questions risks undermining both the implementation of trust-building guidelines and their success. What is needed is a clear understanding of trust linked to a robust economic assessment of trust building activities. Furthermore, any economic assessment of the costs and benefits should acknowledge that different stakeholders, such as investors, industry participants, policy makers, or end users, will prioritize these impacts differently.

In this article, we offer direction on how to close this gap by improving the conceptual understanding of trust, implementing actionable trust performance indicators, and sharpening the trust discourse. While the importance of trust in DHI is commonly recognized, we argue that we still need more evidence-based policies to ensure the trustworthiness of these programs are implemented efficiently and effectively. With this viewpoint, we aim to initiate further development of economic thinking in trust building. More specifically we aim to transfer and adapt knowledge from economic research to other domains where trust building matters, but economic considerations have not yet entered the professional discourse and implementation practice of trust building activities.

## Shortcomings and Remedies

Trust has been part of economic research for centuries [11]. Ample research exists investigating the relationship between trust and societal prosperity [12], the causal impact of trust on economic development [13,14], and how trust lowers transaction costs [15]. As Arrow [16] stated in 1972, “Virtually every commercial transaction has within itself an element of trust, certainly any transaction conducted over a period of time. It can be plausibly argued that much of the economic backwardness in the world can be explained by the lack of mutual confidence…”

While the existing research provides arguments for the economic incentives for trust-building, the costs and benefits of trust-building in digital health have not been well studied. In some emerging economic evaluation guidelines tailored to DHIs, trust can be a component of an impact inventory in user experiences or considered as a non–health-related impact [17]. Yet, trust as an outcome variable is not a well-established component of economic evaluation of digital health. We argue that the lack of focus on the economics of trust-building in this domain include conceptual and methodological challenges that need to be addressed.

## We Need Conceptual Clarity of Trust in Digital Health Interventions as a Prerequisite for Economic Evaluation

Embedded in wider sociopolitical as well as cultural norms and values, the concept of trust is complex and influenced by the context in which it evolves. No commonly accepted definition of trust exists. We suggest that trust in a person or institution is a belief about the trustworthiness of that individual or entity. It is a belief that makes one willing to take a risk and make oneself vulnerable, to rely on that person or institution for one’s future benefit. The trustworthiness trait is defined as having characteristics that are conducive to cooperation. What traits count as trustworthy differ by the situation. Still, we can categorize them as either traits relating to competence (the ability to fulfil the tasks expected of them) and traits related to integrity*,* the commitment to prioritize the best interests of the other [11,18].

Trust is volatile, and while the set of trust-building principles might remain unchanged over time, the relative importance of individual principles can vary depending on the context. In particular, trust repair after health system scandals or negative shifts in societal sentiments toward the government require different approaches [19]. The trait of trustworthiness, often the main target of guidelines, should not be conflated with trust. Trustworthiness is a precondition of trust-building but not a guarantee for trust. Trust-building can be based on calculated decisions and rational thinking [20]. At the same time, a body of literature highlights the importance of emotions in building trust [21]. Combining calculated and emotional motivations to establish trust broadens the spectrum of possible measures available to policy makers and health system actors. Trust research and guidelines in the digital health domain tend to neglect the emotional motivations of trust-building.

Many guidelines contain a comprehensive portfolio of trust-building principles which reflect the overall complexity of the topic but also reflect the lack evidence about which principles work best in practice. Without considering economic constraints and trade-offs, the guidelines may be biased toward presenting too many trust-building principles that are too broad for fear of missing out, with too little guidance on how to prioritize them. As a result, the guidelines may be perceived by users as overwhelming due to the number of principles included or to their high abstractness, with too much onus placed on the implementer to figure out how to navigate the guidelines.

On the receiving end of the guidelines, we see discussions emerging around which organizational units are most suitable to take on the implementation task. The broad spectrum of trust-building principles, from user centered communication and IT security measures to adherence to the rule of law, places responsibility on different organizational units to lead the implementation of guidelines in areas as diverse as public relations, law, compliance, and IT services. Table 2 provides handpicked examples of specific trust-building activities recommended by the guidelines presented in Table 1 that we categorize about accountability for past mistakes, transparency about the present, and promises and benchmarks for the future [11].

**Table 2:** Examples of what trustworthiness guidelines call for.

Guideline	Example trust-building activities according to this guideline		
	Accountability for past mistakes	Transparency in the present	Promises and benchmarks for the future
Ethics guidelines for trustworthy AI^a^ [5]	Mechanisms should be put in place to ensure responsibility and accountability for AIa systems and their outcomes. Auditability, which enables the assessment of algorithms, data and design processes plays a key role therein, especially in critical applications. Furthermore, adequate and accessible redress should be ensured.	The data, system and AIa business models should be transparent. Traceability mechanisms can help achieve this. AIa systems and their decisions should be explained in a manner adapted to the stakeholder concerned. Humans need to be aware that they are interacting with an AIa system and must be informed of the system’s capabilities and limitations.	Adopt a Trustworthy AIa assessment list when developing, deploying or using AIa systems. Adapt it to the specific use case in which the system is being applied.
Digital Trust Framework [6]	Good accountability and oversight mechanisms can both remediate any harms that result from technology use. Auditability serves as a check and is particularly useful in both the development stage and in the deployment stage. Redressability allows for recourse to address any harms. Good accountability builds up the trust necessary for more widespread adoption of useful technologies.	Information is shared about how the technology is developed, implemented and how the data are used. Design is user-friendly with transparency in mind.	Adopt best practice frameworks for security and reliability norms. Digital trust programs that account for the impact of societal expectations.
Global strategy on digital health 2020-2025 [7]	Develop an ethics framework to strengthen public trust.	Use of digital health trust based on authentication and authorization that guarantees trust.	Build trust by placing importance on quality, safety and ethical considerations facilitate evidence-based decisions to improve public trust.
Guidance on the rights-based and ethical use of digital technologies in HIV and health programs [8]	Establish impartial, effective accountability and oversight mechanisms for breaches of data privacy and other rights violations.	Be consultative and transparent in decision-making related to governance and management of digital technologies. Be transparent about and accountable for the factors that influence algorithmic decisions. Co-design digital technologies and systems with affected communities. Create opportunities for digital rights literacy for communities and individuals to understand their rights and take ownership of their data, including the right to withdraw their data from use and to data portability.	Create monitoring and evaluation systems that allow the technologies to adapt based on feedback.
A guide to good practice for digital and data-driven health technologies [9]	Undertake ethical examination of data use specific to this use-case.	Be fair, transparent and accountable about what data is being used. When building an algorithm, show it clearly and be transparent of the learning methodology (if any) that the algorithm is using.	Absent
Code of Ethics for Data-Based Value Creation [10]	If your product depends on data from other companies, make sure that this data has been collected in an ethical manner. Do not use data from untrusted sources.	Only acquire data from partners who are transparent about both their data collection practices, and any restrictions associated with the use of the data. Make it clear to your customers how their data is protected and how access is logged. If it is necessary to store data for a very long time (eg, for legal reasons), explain this to your customers at the time of data collection.	Absent

^a^AI: artificial intelligence.

The intersection between digital health and trust is somewhat consistent regarding accountability for past mistakes and transparency about the present. Benchmarks for the future are less well-articulated across specific trust building activities [7-9], or their scope is limited.

The choice of who within an organization is responsible for the management and implementation of specific guidelines has implications for how financial resources get allocated and how the guidelines get interpreted. A joint effort across different units may be preferable as different trust-building principles require collaboration between different organizational units. Research suggests that a collaborative approach involving a range of actors across the organization enhances trust-building [22].

In response to these requirements, we need implementation evidence. Systematically collected evidence, case studies, and experience reports can inform how resources are allocated for different trust-building principles and identify successful trust-building mechanisms in a given context. Given the context specificity of trust, this evidence will not be a blueprint that can be copied and pasted across contexts. Instead, the evidence can help disseminate experience from comparable settings and is an essential step toward better informed resource allocation.

## We Need Actionable Trust Performance Indicators to Collect Meaningful Evidence

To gather better evidence to inform resource allocation and make judgments about cost effectiveness, we need appropriate methods to collect data about trust-building from the implementation to the evaluation phases. The standard methodology to gather evidence about trust in the context of health care and medicine is survey instruments. Unfortunately, studies have repeatedly shown that the existing instruments have weaknesses. Goudge and Gilson [23] called for advancements in the quantification of trust; Ozawa and Sripad [24] showed in their review of 45 trust measures in health systems that half of the measures used qualitative methods in the design process, and only 33% were pilot tested; Aboueid et al [25] found in a meta-analysis of 26 publications on trust in health professionals and health systems, 10 studies did not mention the dimensions explored and of those that did, some did not define them. Taylor and colleagues [1] call for conceptual clarity and methodological creativity after reviewing 50 years of trust research. Considering the stagnation in methodological development in the field and the increasing inclusion of guidelines regarding trust in digital health, we see a need for refined methods to address questions of cost effectiveness and resource allocation.

An understanding of the resources invested, and outcomes gained is essential. Since we cannot pin a price tag on trust, we need to agree on sensible indicators that we understand as evidence informed. Table 3 gives an overview of currently applied indicators regarding trust in DHIs that have been developed by some of the essential stakeholders in the field, such as the World Bank or WHO. These indicators are the result of a systematic search on indicators to measure digital health system maturity conducted by Maaß et al [26]. The review identified that trust toward digital health applications, AI, the government, and health care providers plays an essential role among assessment tools for digital health system maturity. However, the indicators rarely reported their methodology and data source, thereby limiting their applicability. We require well designed indicators with transparent methodology and a clear definition on what is meant by “trust.”

**Table 3:** Currently used indicators on trust in digital health system interventions.

Indicator	Data source	Reference
Number of individuals identifying false or unreliable information, such as untrustworthy sources, fake news, or fraudulent emails	Quantitative national Computer assisted telephone interviewing survey of 1000 adults aged 18 years or older	Accenture Digital Index by Accenture [27]
You can recognize what information or content online may, or may not, be trustworthy (eg, fact checked information, “fake news” or assess the trustworthiness of a company based on customer reviews)	Qualitative telephone interviewing survey among 4172 adults aged 18 years or older living in the United Kingdom	UK Consumer Digital Index by Lloyds Bank [28]
Regulations and ethical frameworks in place to implement AI^a^ in a way that builds trust and legitimacy	UNCTAD Data Protection and Privacy Legislation Worldwide data overview	Government AI^a^ Readiness Index by Oxford Insights [29]
To what extent do you trust the information you receive from government websites/apps online?	4910 respondents from 98 countries	Inclusive Internet Index by Economist Impact and Meta [30]
To what extent do you trust the information you receive from nongovernment website/apps that are based in your country online?	4910 respondents from 98 countries	Inclusive Internet Index by Economist Impact and Meta [30]
To what extent do you trust that online privacy is guaranteed?	4910 respondents from 98 countries	Inclusive Internet Index by Economist Impact and Meta [30]
Are opportunities available to link data sources safely at the subject level and perform comprehensive analyses – for example, through a closed controlled working environment operated by the statistical office, or through anonymization and linkage by a trusted third party	No data source published	Support tool to strengthen health information systems by the World Health Organisation Regional Office for Europe [31]
Share of population that trusts medical and health advice from the government	Wellcome Trust Global Monitor [32]	Global Health Security Index by the Nuclear Threat Initiative [33]
Share of population that trusts medical and health advice from health professional	Wellcome Trust Global Monitor [32]	Global Health Security Index by the Nuclear Threat Initiative
The number of distinct, publicly-trusted TLS/SSL certificates found in the Netcraft Secure Server Survey	Netcraft and World Bank population estimates	Worldwide Governance Indicators by the World Bank [34]
The share of a population that trusts Artificial Intelligence	Quantitative survey among 62 countries	The Global AI Index by Tortoise [35]
The share of adults in each market saying they trust public bodies using generative AI^a^ technology at least moderately	No data source published	The Network Readiness Index by the Portulans Institute [36]
The trust users hold in institutions, businesses, and policy makers to protect online privacy, harness technology to create jobs, and advocate for consumers	Survey among 42 economies	The Digital Intelligence Index by the Fletcher School at Tufts University [37]
The extent to which institutions create an environment which enables trust	Survey among 42 economies	The Digital Intelligence Index by the Fletcher School at Tufts University [37]
The level of generalized social trust and trust in science and technology in any given economy	Survey among 42 economies	The Digital Intelligence Index by the Fletcher School at Tufts University [37]

^a^AI: artificial intelligence.

Furthermore, most of the guidelines we identified and discussed in Tables 1 and 2 are not published or sponsored by governments or international institutions, raising the question who’s interest it actually is to understand the economics of trust-building in digital health? Without appropriate performance indicators that show whether trust-building principles are successful, we cannot evaluate trust building mechanisms or make decisions about prioritization. Indicators are considered instrumental in driving health system improvements and for running comparative evaluations across the health system [38,39]. DHI investors and developers have good economic incentives to adopt trust performance indicators as it allows them to make better informed decisions about resource allocation for trust building and allows them to better monitor whether trust building actions are successful. Indicator sets are based on robust conceptual frameworks “that set out the rationale and design principles for an indicator set” [40] with the intention to provide standardized benchmarks for the systematic evaluation and monitoring of quality, performance, and outcomes. Addressing company executives, Deloitte argues that trust itself should be a key performance indicator for companies, as drops in consumer trust can considerably erode the market value of the firm [22]. Van der Schee et al [41] suggest public trust in the health system is an important performance indicator for assessing national health systems. Several other examples of indicators in specific areas of the health system exist, such as in digital health care, which include indicators relating to trust at least at the margins [27,28,30,31,33,35-37,42-46]. Discussion is currently centered around identifying useful performance indicators for trust in DHIs and determining the optimal methods for evidence collection. The crucial task is to navigate conceptual complexity and to accommodate context specificity, ensuring the identification of indicators that are suitable for a diverse range of DHIs while aligning with trust-building guidelines.

Survey measures of trust levels can indicate changing trust levels over time [24]. Such measures are useful for assessing the level of trust before and after implementing trust-building principles. Similarly, qualitative user interviews and broader citizen engagement can provide rich information about perceptions and experiences regarding the trustworthiness of a DHI and the effectiveness of its associated trust-building measures [47]. The use of routinely collected data generated from digital health technologies needs to be harnessed and leveraged for better decision-making around trust-building and should include measures of the user experience at different levels of the system. The evidence should inform both the design phase of trustworthy DHIs and for continual assessment of their safety, effectiveness, and quality assurance [48]. This potpourri of methods allows us to think creatively about meaningful performance indicators for various contexts, actors, and DHIs. A comparable example of a newly developed indicator set is the Digital Public Health Maturity Index [49]. This index provides initial indications of possible architecture for an actionable and stakeholder-supported set of performance indicators in an area that faces the same kinds of problems as trust. Developing a trust index and performance indicators with appropriate methods to collect evidence must be integral to future implementation research on trust-building in digital health.

## We Need to Sharpen the Trust Discourse

The increased use of the word “trust” by politicians, industry, and researchers alike raises the question of whether the use of the word trust is always justified. The inflationary use of trust in the public sphere, as seen in commercials, labelling, news articles, and media campaigns, possibly undermines trust’s value in settings where trust discussions are essential [50]. If we are not cautious, we may see the trust debate in the digital health sphere drift into symbolic actions and “trust washing,” similar to experiences in “green washing” and “ethics washing” [51]. Trust degenerates into a hollow marketing term when health system actors invest resources merely to chase trends without meaningfully engaging with internal trust-building procedures and actions leading to overall trustworthiness.

Trust is vital for adopting DHIs, but it is not a panacea. Health system actors can act to the best of their knowledge, and policy makers can design evidence-informed policy, including incorporating trustworthiness guidelines in their policy designs, but that does not mean the public or users will trust us [52]. Trust cannot be enforced or demanded. The public and the users decide how to assess trustworthiness and where to place their trust [53]. That insight implies that investments in trust-building in the context of DHIs carry the risk of failure.

Even if trust building is successful, it may not result in material benefit. For example, if several alternative health system actors adhere to trustworthiness guidelines and are all judged equally trustworthy, users will likely make choices not based on trust but on convenience, price, accessibility, or other adoption enablers. Trust is not the only reason we engage with DHIs. We might depend on a DHI and, consequently, engage with it because we have no other choice. We might assess the risk of misplaced trust and its associated consequences to be low and, therefore, not really care about trust. We might carryover the trust we have for more comprehensive health system to new DHIs, allowing us to spontaneously engage with the new and unfamiliar, similar to the concept of spontaneous sociability [12]. Such considerations are essential and require those aiming to build trust to assess the context carefully and to understand user motivations.

Realistic expectations about the capabilities of trust-building are critical in deciding which trust-building measures to adopt and how best to efficiently deploy the necessary resources.

## Conclusions

Meaningful guidance for trust-building in digital health must go beyond proposing principles to build trust. We argue that the costs and benefits of trust-building measures are currently missing to a large extent. Health system actors and the research community need trust performance indicators to appropriately collect evidence about resources invested and trust gained in return. We need to be mindful of the present inflated use of the term “trust” in the public sphere when designing new trust building efforts, and to acknowledge that trust cannot be guaranteed. To successfully navigate the complexity of trust-building, we need more engagement in the economics of trust-building in digital health. Economics of trust-building needs to be a deeply integrated and structured part of DHI policy making and health system governance at large to develop sustainable trust building processes while bearing in mind resource scarcity. Furthermore, stakeholders across the health system from regulators, to health organizations, to end users need to have guided access to relevant and comprehensive information about these guidelines in order for meaningful progress to be made.

## Abbreviations

AI: artificial intelligence
DHI: digital health innovation
WHO: World Health Organization
